# Deception research today

**DOI:** 10.3389/fpsyg.2014.00256

**Published:** 2014-03-25

**Authors:** Matthias Gamer, Wolfgang Ambach

**Affiliations:** ^1^Department of Systems Neuroscience, University Medical Center Hamburg-EppendorfHamburg, Germany; ^2^Institute for Frontier Areas of Psychology and Mental HealthFreiburg, Germany

**Keywords:** deception, Concealed Information Test, differentiation of deception paradigm, application, theory

## Introduction

Deception is a complex social behavior which involves a set of higher cognitive functions. Studying this common phenomenon in humans has in all epochs been driven not merely by the wish to understand the underlying framework of cognitive functioning but rather by the ambition to detect deceptive behavior in criminal suspects. Thus, identifying valid indicators of deceptive behavior has always been in the focus of deception research. Such indicators can be defined in terms of specific behavior, physiological correlates, or content of verbal reports. The question of how validly each indicator allows for differentiating truthful and deceptive accounts is inherent in the majority of research efforts in this domain.

Another important aspect concerns the development of deception theory. According to current opinion, deception is not characterized by a single cognitive process but rather involves the combination of a variety of basic cognitive processes such as working memory, response monitoring and inhibition. Identifying these processes, modeling their interplay and their modulation by personality and situational factors is still one major challenge in deception research. Furthermore, deception is no unitary phenomenon. Correspondingly, researchers need to examine and describe different variants of this phenomenon occurring in distinct contexts, which entails a variety of experimental and theoretical approaches that largely differ in scope and methods.

## Current interests

One major field in deception research concerns the use of psychophysiological methods to detect deceptive behavior. Over time, the traditional physiological measures (electrodermal, cardiovascular, and respiratory responses) have been supplemented by electroencephalographic, functional imaging, and other innovative procedures. Finding measures that validly discriminate between truth and lie, and the wish to optimize their use, have received new impetus from recent technological development. Neuroimaging techniques, for example, yield new promises and deserve a deep evaluation. Thermal imaging and eyetracking are other innovative methods which might provide additional information about the mental processes involved in generating deceptive responses. However, even “classic” behavioral measures such as response times are still frequently used in this domain for theoretical as well as applied purposes.

Different techniques for detecting deception with the help of physiological measures have been controversially discussed in the scientific community. Among the most influential experimental paradigms, the so-called Concealed Information Test (CIT, Lykken, 1959) has received broad scientific attention. The CIT does not target at identifying deception *per se* but rather aims at detecting whether a suspect has concealed knowledge of specific (e.g., crime-related) details. A different approach is the so-called differentiation-of-deception paradigm (Furedy et al., 1988) which follows the aim to identify specific patterns in behavioral or physiological variables that differ systematically between truthful and deceptive behavior. Particularly this latter approach has been newly fueled by brain imaging techniques which claim to mirror mental processes accompanying deceptive responses more directly.

The current Research Topic brings together contributions from experimental psychology, psychophysiology, and neuroscience focusing on the understanding of the broad concept of deception including the detection of concealed information, with respect to basic research questions as well as applied issues. Due to the interdisciplinary focus of this approach, articles were published in Frontiers in Psychology or Frontiers in Human Neuroscience, respectively.

## Current research

Most articles of this Research Topic focus on the detection of concealed information using variants of the CIT. A large body of previous research has documented that perpetrators show larger electrodermal responses, respiratory suppression, as well as heart rate deceleration to crime-related probes (e.g., a murder weapon) as compared to neutral items (e.g., other unrelated weapons). More recently, comparable differences were reported for behavioral responses, specific components of event-related brain potentials, and neurovascular changes in specific brain regions measured by neuroimaging techniques (for a comprehensive review see Verschuere et al., 2011). Under the premise that innocents to not possess crime-related knowledge, the CIT can be used to validly differentiate perpetrators from innocents. Although the CIT has been first described in the middle of the last century (Lykken, 1959), there are still a number of open questions concerning the theoretical background, the validity of new measures, or special applications for specific circumstances of crimes. Some of these questions were addressed by current articles included in this Research Topic.

Several studies focused on event-related brain potentials and demonstrated that ERP components were susceptible to contextual factors such as the personal involvement in a misdeed during encoding (Jang et al., 2013) or the nature of memory tested in the CIT (episodic vs. semantic, Ganis and Schendan, 2013). Furthermore, it was found that depth of encoding modulated electrodermal responses to crime-related details but did not affect P300 responses in the CIT (Gamer and Berti, 2012). A large study with more than 100 participants reported a modulation of ERP components by personality traits regarding sensitivity to moral and social norms as well as cognitive-motivational conflict processing (Leue et al., 2012). Ambach and colleagues failed to find a similar influence of interindividual differences in psychopathy but reported higher detection accuracy of autonomic measures when the CIT procedure included the face of the fictive interrogator and verbal instead of textual question presentation (Ambach et al., 2012). Two further studies explored the validity of novel physiological and ocular measures in the CIT. Park and colleagues successfully used facial temperature in a periorbital region as determined by thermal imaging to detect concealed knowledge (Park et al., 2013); Seymour and colleagues were able to accurately determine hidden knowledge by differences in pupil responses and blink rates (Seymour et al., 2012). Using a slightly different interrogation protocol, Marchak (2013) could show that eye blink measures even allow for differentiating truthful and false intent. These studies collectively demonstrate that a number of behavioral and physiological variables are susceptible to concealed information and therefore allow for detecting individuals with crime-related knowledge. Moreover, several of these studies reported enhanced classification accuracy when combining different indices of concealed information (Ambach et al., 2012; Seymour et al., 2012; Jang et al., 2013).

Besides using new measures or combining different behavioral and physiological indices, it has been suggested to increase cognitive load during the examination to facilitate the detection of liars. Walczyk and colleagues provided a review and a detailed theoretical outline of this approach (Walczyk et al., 2013). Visu-Petra and colleagues explored such strategy empirically by asking participants to carry out a secondary task simultaneously to the CIT. They could show that such interference facilitates the detection of concealed information based on behavioral measures (Visu-Petra et al., 2013). In a seminal study, Meijer and colleagues developed a novel dynamic questioning approach that does not concern individual responses of single examinees but instead the global responsiveness of a group of suspects. Such method was shown to allow for an identification of specific details of a collectively planned mock terrorist attack (Meijer et al., 2013). Finally, Agosta and Sartori (2013) summarized recent studies on the so-called autobiographical Implicit Association Test; this promising new development allows for accurately determining whether an autobiographical memory is encoded in a given suspect. Taken together, these studies document the substantial advancement of current research on the CIT regarding the potential of novel methods as well as situational and personality factors that are modulating the response pattern. Beyond these efforts, Ben-Shakhar (2012) identified a number of open questions regarding practical aspects and outlined future directions for research on the CIT. These issues are highly relevant for the field implementation of the CIT in police investigations. Such procedure, which is currently only adopted in Japan, is discussed in great detail by Matsuda et al. (2012). The vital international research on the CIT along with the large body of practical experience with this technique in Japan holds promise for further implementations of the CIT as an advancement of currently applied deception detection techniques.

One major problem of current techniques is their susceptibility to countermeasures. Thus, guilty examinees might be able to deliberately alter their pattern of responses to appear innocent. Similarly, certain groups of suspects might have less difficulty in lying as compared to others because of frequent lying in general. Two studies in the current Research Topic explored these issues using variants of the differentiation-of-deception paradigm. Increasing the proportion of deceptive as compared to truthful responses led to reduced reaction time differences between truth and lie, which might indicate that lies require less cognitive effort in frequent liars and are therefore more difficult to detect (Van Bockstaele et al., 2012). Hu and colleagues showed that the instruction to selectively speed up deceptive answers along with a short training substantially altered the pattern of response times such that truthful and deceptive responses became indistinguishable (Hu et al., 2012). However, it cannot be generalized from these results that merely emphasizing that an examination aims at detecting deception necessarily reduces lie detection efficacy. By contrast, a study using a variant of the differentiation-of-deception paradigm in conjunction with functional magnetic resonance imaging revealed larger differences between deceptive and truthful answers in the neural activation of different brain areas when participants believed that a lie-detector was activated (Sip et al., 2013). In line with the majority of neuroimaging studies in this domain (Gamer, 2011), activity in the right inferior frontal gyrus was also modulated by deception. This region was frequently supposed to reflect the recruitment of response inhibition processes. However, temporary disruption of the inferior frontal gyrus by means of continuous theta-burst stimulation did not significantly alter the pattern of behavioral responses in a variant of the differentiation-of-deception paradigm (Verschuere et al., 2012). These results thus question the frequently assumed functional role of the inferior frontal gyrus in deception.

Besides exploring specific cues of deceptive behavior in highly standardized situations and with highly standardized interrogation techniques, it also seems interesting to examine deception in more naturalistic settings. For example, Spence and colleagues asked participants to provide relatively unrestricted honest and deceptive accounts of their opinion regarding social issues. For these accounts, speech parameters were extracted and the authors found a significantly reduced speech rate along with increased response latency during deception compared with truth-telling (Spence et al., 2012). In a similar vein, Duran and colleagues examined movement dynamics accompanying deceptive and truthful accounts. Instead of searching for specific discrete cues such as the rise of a brow, they examined the whole time course of movements and provided preliminary evidence for unique dynamic signatures of deception in these kinetic variables (Duran et al., 2013). Finally, Mackinger and Jonas (2012) explored determinants of deception in advisor-client interactions and provided evidence for the use of explicit and implicit strategic deceptive behavior in advisors aiming to receive an incentive.

## Perspective

Research on deception has a long tradition in psychology and related fields. On the one hand, the drive for detecting deception has inspired research, teaching, and application over many decades. On the other hand, research on deception as a process or phenomenon is characterized by manifold interactions with other areas of psychological research such as attention, memory, executive control, or motor behavior. It remains to be debated whether deception and its detection should be studied as a key topic which entails addressing these other fields, or rather as a particular, illustrative manifestation of them. We regard the present Research Topic as clearly underlining the scientific benefits arising from the broad and multidisciplinary perspective that characterizes deception research today and we hope that it will enrich and inspire future research in this domain.
